# Physiology

**Published:** 1860-07

**Authors:** 


PHYSIOLOGY.—Our esteemed collaborator, Dr. R. J. DUNGLISON, has in press an elementary work on physiology, adapted to the use of colleges, academies, and schools, which will embrace the applications of descriptive anatomy, microscopy, and organic chemistry, to the elucidation of the various subjects of which it will treat. From the well-known qualifications of the author, we feel confident that he will execute his labors in an able manner, and that the work will be admirably suited to the purposes for which it is intended.

